# Study of total stimulated saliva flow and hyperpigmentation in the oral mucosa of patients diagnosed with hereditary hemochromatosis. Series of 25 cases

**DOI:** 10.4317/medoral.17206

**Published:** 2011-12-06

**Authors:** Miguel-Angel Sánchez-Pablo, Vanesa González-García, Alejandro del Castillo-Rueda

**Affiliations:** 1 University Diploma in Nursing, Dentistry student, Universidad Rey Juan Carlos, Alcorcón, Madrid; 2 Fellow, Dentistry student, Universidad Rey Juan Carlos, Alcorcón, Madrid; 3 Head of the Iron Disorders Unit, Associate Professor of Health Sciences

## Abstract

Objective: To study lesions in the oral cavity of patients with hereditary hemochromatosis and determine their association with iron overload. Study Design: We took a clinical history, examined the pigmentation of the oral mucosa, and measured total stimulated saliva production. We correlated our results with epidemiological, phenotypic, and genotypic findings. Patients with associated diseases or drug therapy causing xerostomia were excluded. Results: We evaluated 25 patients (20 men, mean age 52 years) over a period of 6 months. No patient complained of xerostomia and pigmentation was not detected in the oral mucosa. The total stimulated salivary flow was reduced in 9 patients who had an average ferritin level of 796.5 μg/l. The decline in total stimulated salivary flow was significantly correlated with ferritin levels (p=0.002). Patients with ferritin levels within the normal range also had normal stimulated salivary flow. Conclusions: We found no pigmented lesions in the oral mucosa; however, we did observe a decrease in total stimulated salivary flow that correlated with ferritin levels. Therefore, hyposialia caused by functional impairment of the salivary glands may be an early marker of iron deposition.

** Key words:** Hemochromatosis, mucosa, mouth, saliva, xerostomia, hyposialia

## Introduction

Hereditary hemochromatosis (HH) is an autosomal recessive disease characterized by increased intestinal absorption of dietary iron, which progresses to overload and slow and progressive deposition of iron in various organs. This in turn leads to tissue damage with structural abnormalities (fibrosis) and functional abnormalities (cirrhosis, cardiomyopathy, diabetes, arthropathy, osteoporosis, skin pigmentation, and hypogonadism) (1). However, diagnosis of HH is increasingly based on laboratory findings and not on clinical criteria. Furthermore, in asymptomatic patients or patients with nonspecific symptoms such as fatigue or ar-thralgia, hyperpigmentation is not generally an early clinical sign (2). Oral manifestations are not usually recorded as signs or symptoms, especially if no skin hyperpigmentation is present (3). The absence of specific clinical manifestations appears to be more related to early diagnosis, the presence of iron overload (mild or moderate), and the absence of serious complications such as cirrhosis. Skin and mucosal manifestations have been described in highly developed clinical forms (1). 

The classic clinical presentation of HH with iron overload is associated with mutations in the HFE gene, namely, C282Y and/or H63D. C282Y is more common in northern Europe; H63D is more common in southern Europe, including Spain, and causes less severe disorders. In addition to the primary clinical or genetic form, secondary iron overload can be observed, mainly in patients undergoing multiple transfusions, with clinical manifestations that sometimes overlap with those of HH (4). Although also an acute phase reactant, serum ferritin is used to assess iron stores in the body; higher values indicate iron overload and can be used to assess associated risk (2). Thus, lesions of the oral mucosa and alterations in salivary flow (5, 6) have traditionally been studied in patients with elevated iron deposits, severe organ involvement (mainly the liver), and skin hyperpigmentation, with high ferritin values indicating severe iron overload (7-9). We studied manifestations in the oral mucosa and salivary flow in patients with HH in a unit dedicated to early diagnosis and genetic counseling of patients with this disease. Related disorders of the oral cavity were examined in terms of organ involvement, genotype, and degree of iron overload. The objectives of this study were to detect the presence of pigmentation in the oral mucosa of patients with HH and to determine whether there was a relationship between levels of iron overload and total stimulated salivary flow (SSF) (5-7).

## Material and Methods

This prospective cross-sectional study was conducted from October 2009 to March 2010. The study sample comprised patients diagnosed with HH in the Iron Disorders Unit of Hospital General Universitario Gregorio Marañón (Madrid, Spain) and first-degree relatives of these patients. We excluded patients with associated diseases and/or those receiving medication that could alter salivary flow (10). 

The study was designed and conducted in accordance with the principles of the Declaration of Helsinki (2008) and local ethics requirements. Prior to implementation, all patients gave their informed consent to participate. The pigmentation of the oral mucosa was examined and total SSF was determined using sialometry. We collected epidemiological data and data on general and specific oral symptoms. Patients who replied positively to the question “are you normally aware of your dry mouth?” were considered to have xerostomia (11,12). We evaluated the presence of associated diseases, general and specific drug therapy that could potentially cause xerostomia, phenotype (ferritin levels), and genotype (study of C282Y and H63D in the HFE gene). The physical examination revealed the condition of the labial, lingual, gingival, and buccal mucosa. SSF was assessed by asking the patient to chew on a piece of paraffin for one minute. The patient then swallowed the paraffin and the saliva generated during the following five minutes was collected and measured using a stopwatch and a pipette (13). The patient was given general instructions and asked not to eat, smoke, or chew gum during the 90 minutes before the test. The same person collected saliva samples under the same conditions between 12:00 and 14:00 to minimize the influence of circadian rhythms (14). Due to the large variation in SSF between subjects, it is difficult to establish the cutoff for normal values. However, 1 to 2 ml/min is generally considered a normal rate (15,16). Hammarén and Hugoson (17) observed a reduction in SSF, with an average of 0.9 ml/min. In our study, reduced SSF was considered to be that falling below the first tertile (<0.8 ml/min). The statistical analysis was performed using SPSS ® 16. The Mann-Whitney test was applied for two independent samples. Statistical significance was set at ≤0.05.

## Results

**Figure 1:**
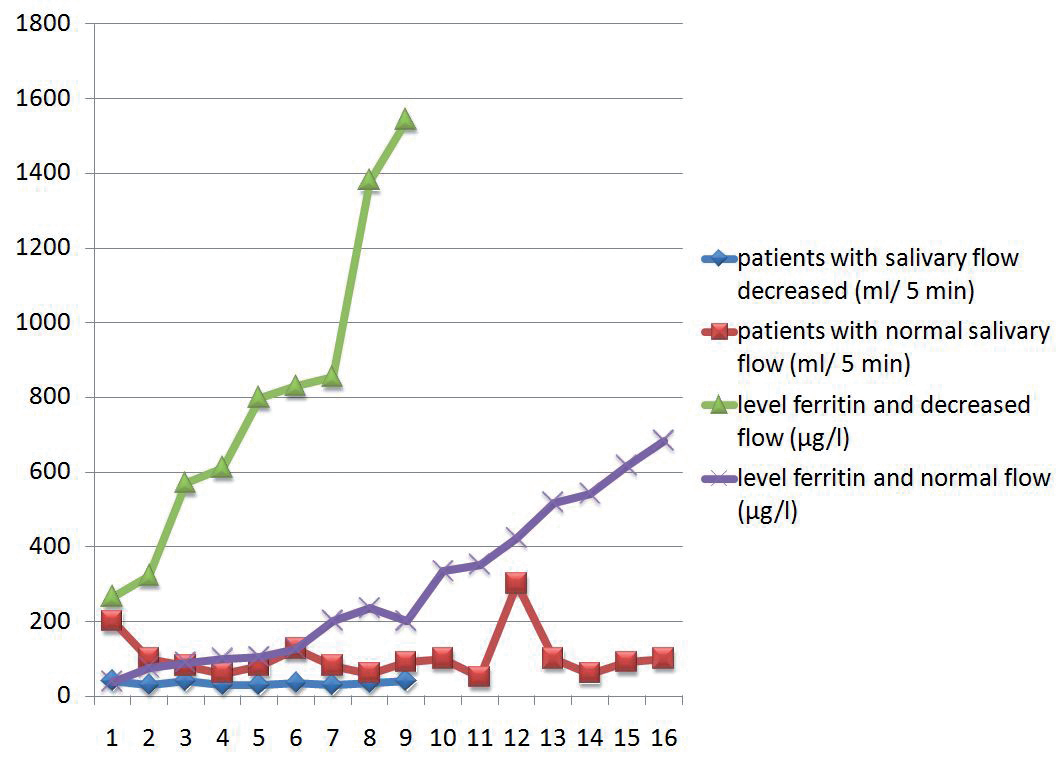
Salivary secretion and normal and decreased ferritin levels.

The study sample comprised 25 patients (20 men; mean [SD] age, 52 [13.1] years), none of whom reported suffering from xerostomia. Pigmented lesions were not detected in the oral mucosa in any patients. The mean (SD) ferritin level was 475.4 (387.5) µg/l (normal range 26-370 μg/l), ranging from 38 to 1543 ( Table 1). Mean total SSF was 1.5 (1.2) ml/min. Decreased flow was detected in 9 patients (36%). Patients with reduced SSF had ferritin levels above 266 µg/l (mean, 796.5 µg/l) and a mean SSF of 0.68 ml/min (Fig. 1). In patients with a normal salivary function, we observed a flow of 2 ml/min, and a mean level of ferritin 294.7 µg/l ( Table 2). All patients with normal ferritin levels had a preserved SSF. 

**Table 1:**
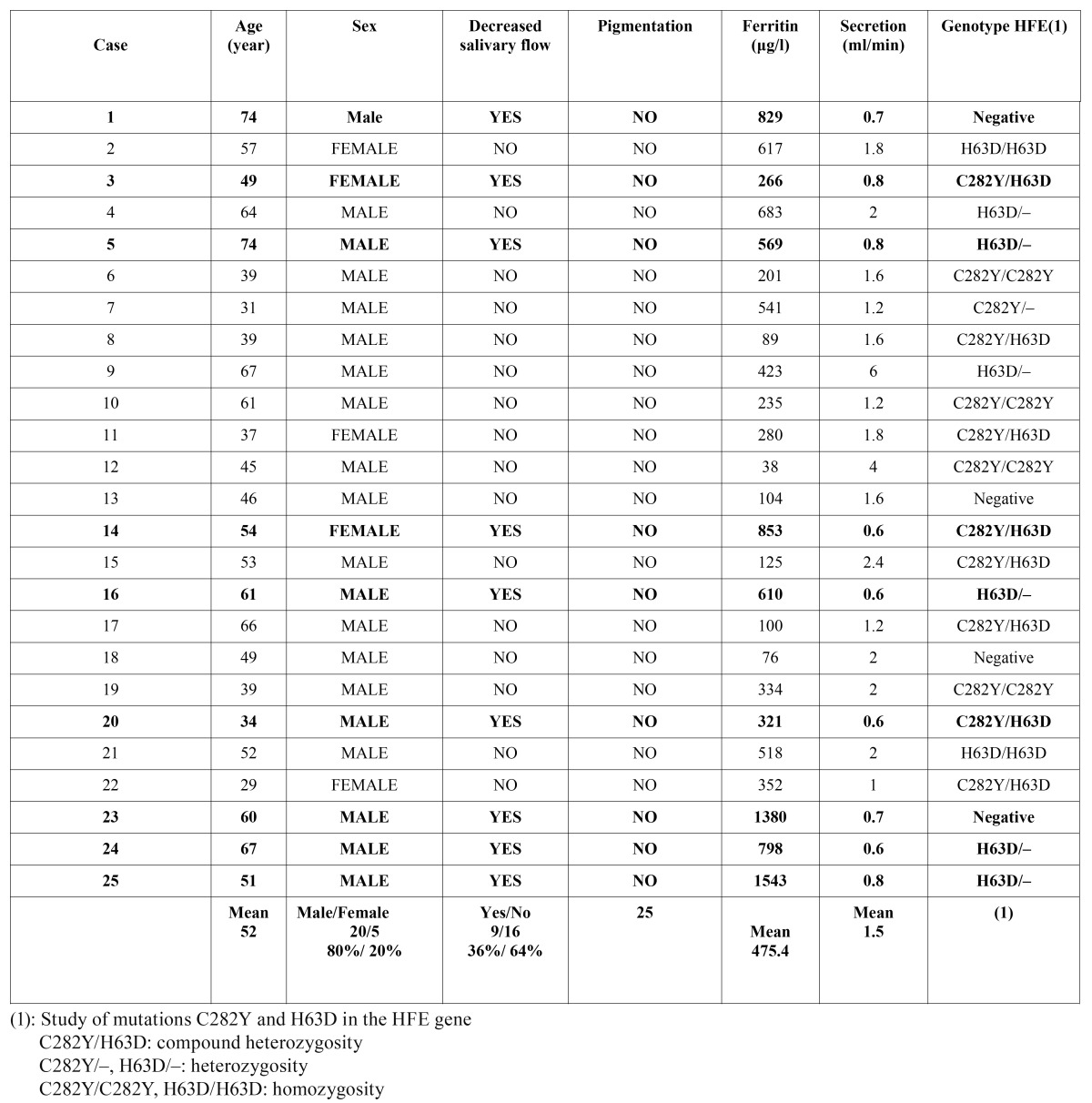
Characteristics of the 25 cases of hereditary hemochromatosis.

Analysis by gender revealed a mean age for men of 53 (12.8) years, a ferritin level of 501 µg/l and a total SSF of 1.7 ml/min. Seven men had a mean ferritin level of 864.2 µg/l, with a mean flow of 0.68 ml/min. Patients with normal total SSF had a mean ferritin level of 306.4 µg/l, and a mean flow of 2.2 ml/min ( Table 2). The mean age of the 5 women studied was 47 (14.4) years. Ferritin level was 370 µg/l, and salivary flow 1.08 ml/min. Two women had hyposialia (559.5 µg/l), with a discharge of 0.7 ml/min. Patients with normal salivary flow had a ferritin level of 244 µg/l and a discharge of 1.3 ml/min. 

We compared mean ferritin levels in patients with hyposialia with those of subjects with normal salivary flow using the nonpara-metric Mann-Whitney test (the sample was smaller than 30 individuals). The difference was statistically significant (p = 0.002) ( Table 2). The study of the C282Y and H63D mutations in the HFE gene in 9 patients with iron overload and hyposialia revealed 4 patients with a heterozygous genotype H63D and 3 cases with compound heterozygosity C282Y/H63D. The remaining 2 did not show any mutations in the HFE gene ( Table 1).

**Table 2:**
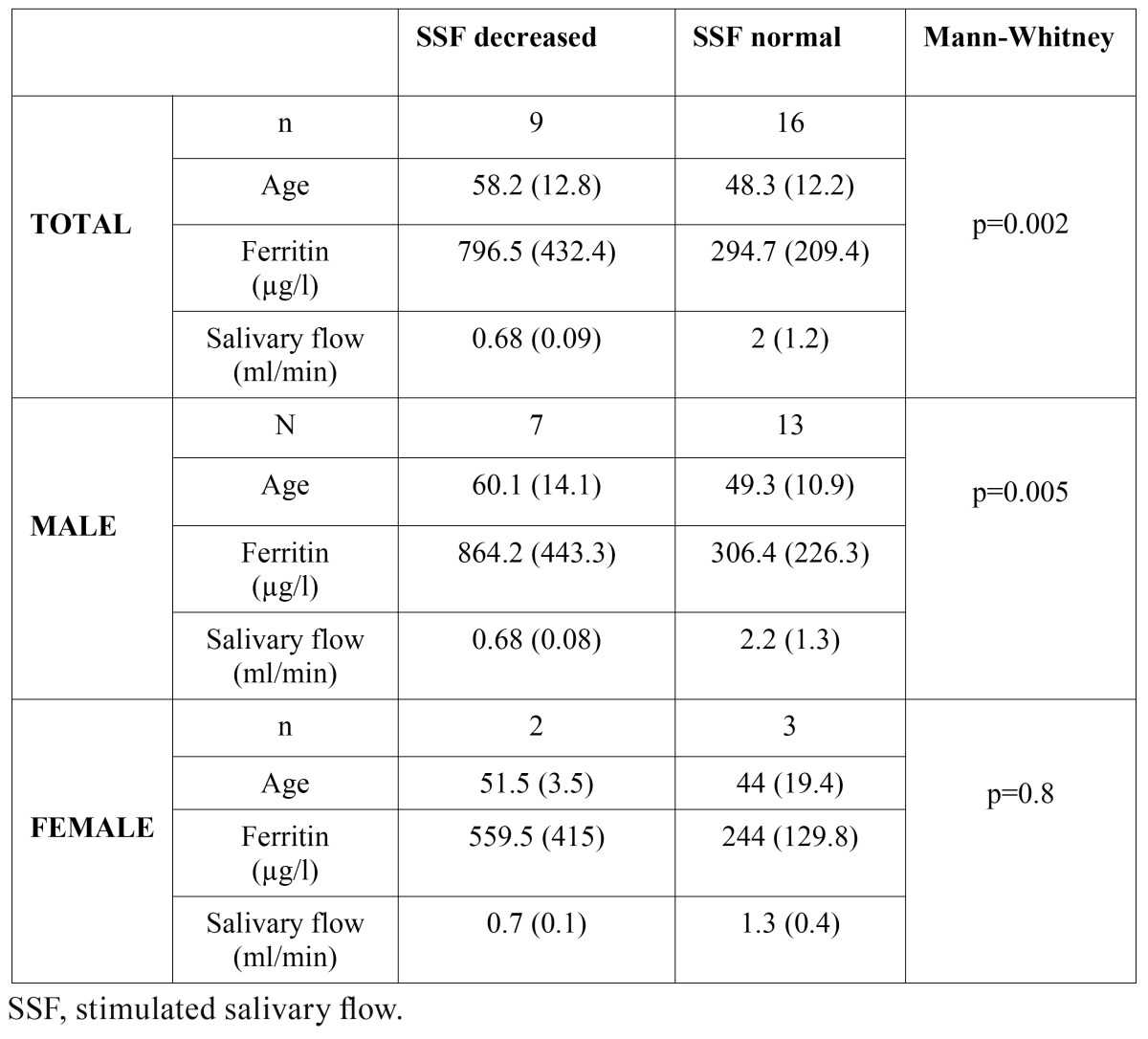
Association between SSF and ferritin level, and sex, expressed as mean (SD).

## Discussion

Our patients showed no pigmentation in the oral mucosa, although the frequency of this finding has been estimated to be between 15% and 20% (8, 9). We attribute this lack of pigmentation to the small sample size and the fact that our patients had mild or moderate iron overload, with no associated diseases. However, in 9 patients, we observed that SSF had fallen, as reported elsewhere (6), in patients with no organic, functional, or pharmacological causes of hyposialia (mainly psychoactive parasym-patholytics, antihypertensives) (10). No patients presented xerostomia, and, in cases in which hyposialia was demonstrated, the most likely cause may be metabolic in origin, namely sialosis caused by iron deposition in parenchymal cells, as reported else-where (6,7). None of our patients had the classic genotype, homozygous C282Y, whereas heterozygous H63D (4 cases) and compound heterozygosis (3 cases) predominated. No mutations were found in the 2 remaining cases. The Mann-Whitney test revealed a p value of 0.002, thus justifying the conclusion that there are significant differences in ferritin levels in patients with reduced and normal salivary flow. Given the limitations of the sample size and the characteristics of patients with mild or moderate iron overload, future studies with larger sample sizes should be performed to assess the outcome of patients without iron overload undergoing phlebotomy and to study whether SSF values return to normal. Therefore, this condition seems to involve a reversible functional impairment that improves after phlebotomy and normalization of ferritin levels. Takeda and Ohya (7) reported a case of hemochromatosis secondary to iron injection and blood transfusion—evident from the iron deposits in the salivary glands—together with xerostomia that disappeared after treatment with deferoxamine (parenteral iron chelator), thus demonstrating the reversibility of the process. If our hypothesis that the decrease in SSF is reversible and related to iron overload is confirmed, we propose that this marker is associated with other markers of iron overload and should be used as a clinical criterion of disease progression.

## References

[1] Pietrangelo A (2004). Hereditary hemochromatosis--a new look at an old disease. N Engl J Med.

[2] Dever JB, Mallory MA, Mallory JE, Wallace D, Kowdley KV (2010). Phenotypic characteristics and diagnoses of patients referred to an iron overload clinic. Dig Dis Sci.

[3] Allen KJ, Gurrin LC, Constantine CC, Osborne NJ, Delatycki MB, Nicoll AJ (2008). Iron-overload-relateddisease in HFE hereditary hemochromatosis. N Engl J Med.

[4] Toxqui L, De Piero A, Courtois V, Bastida S, Sánchez-Muniz FJ, Vaquero MP (2010). Iron deficiency and overload, Implications in oxidative stress andcardiovascular health. Nutr Hosp.

[5] Ward-Booth P, Ferguson MM, MacDonald DG (1981). Salivary gland involvement inhemochromatosis. Oral Surg Oral Med Oral Pathol.

[6] Dean DH, Hiramoto RN (1984). Submandibular salivary gland involvement in hemochromatosis. J Oral Med.

[7] Takeda Y, Ohya T (1987). Sicca symptom in a patient with hemochromatosis: minor salivary gland biopsy for differential diagnosis. Int J Oral Maxillofac Surg.

[8] Rydosz J (1975). Pigmentary changes in oral mucosa. Czas Stomatol.

[9] Frantzis TG, Sheridan PJ, Reeve CM, Young LL (1972). Oral manifestations of hemochromatosis. Report of a case. Oral Surg Oral Med Oral Pathol.

[10] Silvestre-Donat FJ, Miralles-Jordá L, Martínez-Mihi V (2004). Protocol for the clinical management of dry mouth. Med Oral.

[11] Sreebny LM, Valdini A (1988). Xerostomia, Part I: Relationship to other oral symptoms and salivary gland hypofunction. Oral Surg Oral Med Oral Pathol.

[12] Bergdahl M (2000). Salivary flow and oral complaints in adult dental patients. Community Dent Oral Epidemiol.

[13] Iorgulescu G (2009). Saliva between normal and pathological, Important factors in determining systemic and oral health. J Med Life.

[14] Dawes C, Ong BY (1973). Circadian rhythms in the flow rate and proportional contribution of parotid to whole saliva volume in man. Arch Oral Biol.

[15] Wolff M, Kleinberg I (1998). Oral mucosal wetness in hypo- and normosalivators. Arch Oral Biol.

[16] Bertram U (1967). Xerostomia:Clinical aspects, pathology and pathogenesis. ActaOdontol Scand.

[17] Hammarén M, Hugoson A (1989). Clinical psychiatric assessment of patients with burning mouth syndrome resisting oral treatment. Swed Dent J.

